# Electronegative LDL: A Circulating Modified LDL with a Role in Inflammation

**DOI:** 10.1155/2013/181324

**Published:** 2013-08-22

**Authors:** Montserrat Estruch, José Luis Sánchez-Quesada, Jordi Ordóñez Llanos, Sònia Benítez

**Affiliations:** ^1^Servei de Bioquímica, Instituto de Investigaciones Biomédicas Sant Pau (IIB Sant Pau), 167 Sant Antoni Maria Claret, 08025 Barcelona, Spain; ^2^Departament de Bioquímica i Biologia Molecular, Universitat Autònoma de Barcelona, 08193 Barcelona, Spain; ^3^Servei de Bioquímica, Hospital de Sant Pau, 08026 Barcelona, Spain

## Abstract

Electronegative low density lipoprotein (LDL(−)) is a minor modified fraction of LDL found in blood. It comprises a heterogeneous population of LDL particles modified by various mechanisms sharing as a common feature increased electronegativity. Modification by oxidation is one of these mechanisms. LDL(−) has inflammatory properties similar to those of oxidized LDL (oxLDL), such as inflammatory cytokine release in leukocytes and endothelial cells. However, in contrast with oxLDL, LDL(−) also has some anti-inflammatory effects on cultured cells. The inflammatory and anti-inflammatory properties ascribed to LDL(−) suggest that it could have a dual biological effect.

## 1. Introduction

The inflammatory properties of modified LDLs are a main topic in atherosclerosis research. In addition to their inflammatory properties, modified LDLs are recognized by the scavenger receptor (SR), leading to the formation of lipid-loaded foam cells, typical of atherosclerotic lesions. LDL can be modified in arterial intima and in plasma circulation by several mechanisms, such as glycation, lipolysis, aggregation, and oxidation [1]. Oxidized LDL (oxLDL) and minimally modified LDL (mmLDL), a mild oxidized LDL, are the most widely studied modified LDLs in the literature. The involvement of oxLDL and mmLDL in atherogenesis and inflammation in the arterial wall is well established [2], but they have been detected in blood only at a very low concentration [3].

Electronegative LDL (LDL(−)) is a modified circulating form of LDL found in blood. It is an LDL subfraction with a high negative charge that constitutes about 3–5% of the total LDL in normolipidemic (NL) subjects. Its existence was first reported by Avogaro in 1988 [4]. Numerous studies focusing on LDL(−) have since been performed, and the most widely accepted idea is that LDL(−) is a pool of LDL particles modified by several mechanisms. 

LDL(−) has several physicochemical characteristics that differ from native LDL (hereafter referred to as LDL(+)) [5, 6]. Regarding lipid and protein composition, LDL(−) has a higher content of triglycerides [7], nonesterified fatty acids (NEFA) and lysophosphatidylcholine (LPC) [8], and ceramide (CER) [9] than LDL(+). It also shows associated phospholipolytic activities that are absent in LDL(+) [10, 11]. LDL(−) has an abnormal apolipoprotein B (apoB) conformation, which seems to play a role in both its greater binding to proteoglycans (PG) and greater susceptibility to aggregation than LDL(+) [12]. These physicochemical properties are likely responsible for its biological effects in different cell types that participate in the atherosclerotic process. 

Early studies regarding the biological effects of LDL(−) were performed in endothelial cells. It was found that LDL(−) promoted cytotoxicity [13, 14] and release of inflammatory cytokines [7]. The cytokine release effect has since been reported in monocytes and lymphocytes [15]. These observations support an atherogenic role for LDL(−). Nevertheless, recent data suggest that LDL(−) may not only have such an inflammatory role as was first thought. Studies in mononuclear cells have shown that LDL(−) has the ability to induce anti-inflammatory cytokine IL10 [15] and counteract the inflammatory effect promoted by lipopolysaccharide (LPS) [16].

This review focuses on the biological effect of LDL(−) on cells, emphasizing its role in monocytes, which are pivotal to the inflammatory response in atherosclerotic lesions. We discuss the dual function of LDL(−), inflammatory and anti-inflammatory, and its physiological role. 

## 2. A Heterogeneous LDL

Although LDL(−) was first considered an oxidized particle in the circulation, it is now widely accepted to be a pool of modified LDLs with different properties but sharing the common feature of increased electronegativity. Nowadays, LDL(−) heterogeneity is considered a consequence of its different origins.

The oxidative origin of LDL(−) is controversial. Avogaro et al. and Sevanian et al. reported that LDL(−) has a lower vitamin E content [17], a higher amount of lipoperoxides and oxidized cholesterol [14, 17], and a higher susceptibility to oxidation [18] than LDL(+). However, other studies do not replicate these findings [19, 20]. Chen and coworkers focused their research on the most electronegative LDL subfraction, the so-called L5, detected in dyslipidemic patients but not in NL subjects [21]. They described that L5 is a mild oxLDL subfraction contained in the whole pool of LDL(−). The observation that L5 is a minor LDL(−) subfraction is in agreement with the oxLDL proportion found in blood (0.1–0.5% of total LDL) [3] compared to the LDL(−) proportion (3–5%) [5].

It has been suggested that LDL modifications other than oxidation contribute to the generation of LDL(−). Such modifications include nonenzymatic glycosylation, NEFA enrichment, and modification by phospholipolytic enzymes: phophospholipase A2 (PLA2) and sphingomyelinase (SMase) [1]. These modifications are known to increase the negative charge of LDL and likely to occur not only in blood but also in the arterial intima. It is described that in the arterial intima of atherosclerotic lesions there is an overexpression of PLA2 and SMase [22, 23], which could generate LDL(−). 

LDL(−) is heterogeneous in size and density. This heterogeneity seems to depend on the mechanism involved in the generation of the particle. LDL(−) are small-dense particles in NL subjects and large-buoyant particles in familial hypercholesterolemic (FH) subjects, whereas hypertriglyceridemic patients can present both dense and light particles [24]. 

LDL(−) is also heterogeneous in its lipid and protein content. Compared to native LDL, it has an increased content of several non-apoB apolipoproteins: apoE, apoCIII, apoAI, apoAII, apoD, apoF, and apoJ [25]. Besides apolipoproteins, LDL(−) has a higher content in platelet-activating factor acetylhydrolase (PAF-AH) than LDL(+), leading to an increase in its enzymatic activity. Another enzymatic activity found in LDL(−) is the phospholipase C (PLC)-like activity [11]; its origin in LDL(−) is unknown, and it is absent in LDL(+). Both enzymatic activities in LDL(−) could be responsible for the altered lipid content in LDL(−), including its higher content in NEFA, LPC, and CER than LDL(+). These three lipid components are related to the inflammatory effect of LDL(−) on cultured cells [8, 9, 26]. The increased NEFA and LPC content in LDL(−) seems to be generated by hydrolysis of choline-containing phospholipids by PAF-AH activity [5] and the increased CER content by hydrolysis of sphingomyelin by PLC-like activity [9]. 

Finally, the heterogeneity of LDL(−) is also suggested by the presence of a minor proportion of an aggregated subfraction (agLDL(−)). AgLDL(−) seems to be responsible for the PLC-like activity of LDL(−), since such activity is mainly present in agLDL(−) [27]. It has been described that the heterogeneity in the aggregation level is responsible for LDL(−) populations with a normal or high binding affinity to PG compared to native LDL [12]. A relationship between aggregation and the abnormal apoB conformation of LDL(−) also exists [12].

## 3. An Atherogenic LDL 

**Figure 1:**
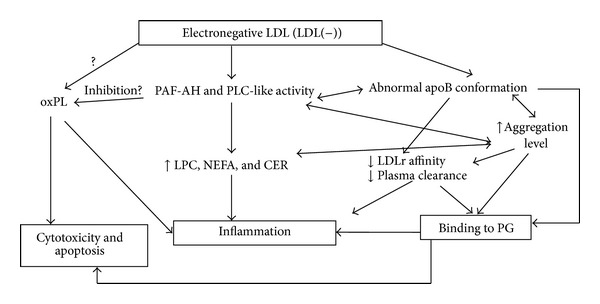
Putative relationships between the physicochemical properties of LDL(−) and its inflammatory actions. Phospholipolytic activities contained in LDL(−) increase its LPC, NEFA, and CER content. These compounds are involved in the inflammatory action of the particle. Phospholipolytic activities could also be related to the abnormal apoB conformation and high aggregation of LDL(−), which may contribute to its decreased plasma clearance and increased binding to PGs. The retention of LDL(−) to endothelium by PG would favor the inflammatory action of LDL(−) on the arterial wall cells. Some authors have suggested that the presence of oxPL in LDL(−) is responsible for the inflammatory, cytotoxic, and apoptotic effects of this particle. LDL(−): electronegative LDL, oxPL: oxidized phospholipids, PAF-AH: platelet-activating factor acetylhydrolase, PLC: phospholipase C, LPC: lysophosphatidylcholine, NEFA: nonesterified fatty acids, CER: ceramide, apoB: apolipoprotein B, LDLr: LDL receptor, PG: proteoglycans.

Several inflammatory effects have been ascribed to LDL(−), and they are probably a consequence of the combination of the different LDL(−) physicochemical properties (Figure 1). These inflammatory effects and other evidence described in this section suggest that this modified LDL could play an atherogenic role and be a putative biomarker of cardiovascular risk, as suggested elsewhere [28, 29]. The usefulness of LDL(−) as a biomarker in the diagnosis of cardiovascular risk should be determined in large cohorts of patients, but methods to do this are still under development [28]. 

### 3.1. Increased LDL(−) Proportion in Inflammation

The first evidence of the relationship between LDL(−) and atherosclerosis is the increased proportion of LDL(−) in subjects with pathologies known to be associated with cardiovascular risk and inflammation. These pathologies include FH [30], hypertriglyceridemia [24], type 1 and type 2 diabetes mellitus (DM) [31, 32], chronic kidney disease requiring hemodialysis [33, 34], and rheumatoid arthritis [35]. LDL(−) is also increased in patients with acute myocardial infarction [36] and angiographically documented coronary artery disease [37]. In each pathology, the mechanisms involved in LDL(−) generation likely depend on the individual characteristics and the underlying disease of the patients. Some drugs administered to treat DM and FH, such as insulin and statins, decrease the proportion of LDL(−), besides decreasing the cardiovascular risk [30, 32]. 

Moreover, a high LDL(−) proportion has been associated with a worse lipid profile since there is a positive correlation of LDL(−) proportion with nonhigh density lipoprotein cholesterol (non-HDLc) and a negative correlation with HDLc [38].

### 3.2. Immunological Response Induced by LDL(−)

It has been described that LDL(−) can trigger an adaptative immune response, leading to the production of anti-LDL(−)-autoantibodies and immunocomplexes, which can be quantified by ELISA [39]. The presence of these autoantibodies is increased in DM [40] and in acute coronary syndromes [41]. Grosso et al. reported that anti-LDL(−)-autoantibodies administered intravenously in mice can play a protective role in atherosclerosis [42]. Taken together, it seems that anti-LDL(−)-autoantibodies could be useful biomarkers in patients with high risk for coronary events [39, 41].

### 3.3. Apoptotic and Cytotoxic Effects of LDL(−)

Some authors have reported that LDL(−) has cytotoxic properties in cultured endothelial cells. This was considered due to its high content of oxidized cholesterol [14, 43]. In contrast, other authors have reported that LDL(−) has no cytotoxic effect [7, 15] or that its cytotoxic effect is due to mechanisms other than oxidation [13]. The divergence in results is probably a consequence of the LDL(−) heterogeneity. 

There is an agreement that LDL(−) induces apoptosis. Chen and colleagues reported that the highly electronegative LDL subfraction L5 promoted apoptotic effects on endothelial cells through a decrease in fibroblast growth factor 2. This induction of apoptosis was found for L5 isolated from FH [44, 45], DM [46, 47], and smokers [48]. The apoptotic effect was suppressed in the presence of low concentration of aspirin [36]. These authors attributed the apoptotic ability of L5 to oxidation. However, the apoptotic effect could be due to the increased CER content in LDL(−) since CER is an inductor of apoptosis [49]. An apoptotic effect of LDL(−) was also shown in macrophages [50] and in cardiomyocytes [51]. In the latter study, it was found that apoptosis was induced by culture-conditioned medium of endothelial cells incubated with LDL(−). In addition, LDL(−) has been described to induce in lymphocytes and macrophages the gene expression and membrane-bound protein of Fas [50, 52], a factor that triggers extrinsic pathway of apoptosis [53]. 

At subapoptotic concentrations, however, L5 impairs differentiation of endothelial progenitor cells and inhibits endothelial cell regeneration and neovascularization [48]. In endothelial cells, L5 also inhibits reendothelization [46], growth, and survival signaling [54] and activates cell stress by promoting inflammation and mitochondrial dysfunction [55].

### 3.4. Inflammatory Properties of LDL(−)

**Figure 2:**
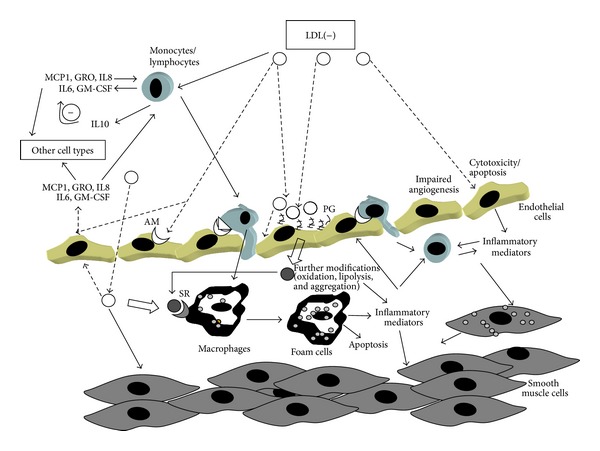
Biological actions of LDL(−) on circulating mononuclear cells (monocytes/lymphocytes) and arterial wall cells (endothelial cells, macrophages and smooth muscle cells) in relation to atherogenesis. LDL(−) can activate circulating leukocytes, mainly monocytes, and lymphocytes. LDL(−) also induces chemokine and adhesion molecules in endothelial cells, promoting the recruitment of more circulating leukocytes to endothelium. Cytokines released by endothelial cells can also act on other cell types of the arterial wall. LDL(−) retained in the subendothelial space by its increased binding to PG can also stimulate arterial wall cells. In this environment, LDL(−) could be further modified, leading to additional inflammatory actions on cells. It could also be uptaken by SR, promoting the formation of foam cells. LDL(−): electronegative LDL, MCP1: monocyte chemoattracting-protein 1, GRO: growth-related oncogen, IL6, IL8, and IL10: interleukin 6, 8, and 10, GM-CSF: granulocyte monocyte-colony stimulating factor, SR: scavenger receptor, AM: adhesion molecule, PG: proteoglycans.

There is consensus that LDL(−) induces an inflammatory response on cells participating in the atherosclerotic process. The most important effect induced by LDL(−) is the release of cytokines, particularly in endothelial and mononuclear cells.  Figure 2 summarizes the role of LDL(−) in atherogenesis in relation to the inflammatory effects promoted on cells.

#### 3.4.1. Effects on Endothelial Cells

The endothelium is the physical barrier between blood and the vessel wall. Endothelial cells control important physiological processes, including cellular trafficking. They also control the recruitment of circulating monocytes and lymphocytes to the arterial endothelium. Infiltration of these circulating cells to sites of inflammation is one of the earliest events in atherosclerosis. It has been described that LDL(−) attracts monocytes and lymphocytes to endothelial cells [21, 56], suggesting its participation in the early phases of atherosclerosis. It has been reported that LDL(−) promotes this attraction by inducing adhesion molecules and chemokine release in endothelial cells. In relation to adhesion molecules, LDL(−) induces vascular cell adhesion molecule (VCAM) [56, 57]. The induction of chemokine release by LDL(−) was first reported by De Castellarnau et al. who observed that LDL(−) promotes monocyte chemotactic protein 1 (MCP1) and interleukin 8 (IL8) release in human umbilical vein endothelial cells (HUVEC) [7]. MCP1 and IL8, respectively, induce the recruitment of monocytes and T lymphocytes to the endothelium. The release of these chemokines in HUVEC has been reported for LDL(−) isolated from NL [7], FH [20], and DM subjects [58]. As the LDL(−) proportion is higher in FH and DM than in NL, the inflammatory effect promoted by LDL(−) should be greater in these patients than in NL subjects.

Further studies in HUVEC have shown that LDL(−) induces other inflammatory cytokines, such as interleukin 6 (IL6), growth-related oncogen (GRO), granulocyte-monocyte-colony stimulating factor (GM-CSF) [59], and epithelial cell-derived neutrophil-activating peptide 78 [56]. The cytokine release promoted by LDL(−) has been reproduced in cultured human endothelial cells of arterial origin [60]. In bovine arterial endothelial cells, the most electronegative subfraction L5 also induces secretion of matrix metalloproteinases and vascular endothelial growth factor expression [45].

#### 3.4.2. Effects on Monocytes and Lymphocytes

Besides endothelial cells, lymphocytes and particularly monocytes play a pivotal role in atherogenesis and inflammation by secreting cytokines and growth factors. As they are present in blood, it is highly feasible that they interact with LDL(−). For this reason, the interaction between mononuclear cells and LDL(−) has been a focus for study in recent years. It has been observed that LDL(−) induces the release of the same cytokines in mononuclear cells, monocytes, and lymphocytes, as in endothelial cells [15]. However, LDL(−) induces anti-inflammatory IL10 in mononuclear cells [15], but not in endothelial cells [59]. The putative physiological role of the IL10 production and other theoretically anti-inflammatory actions promoted by LDL(−) will be discussed further on.

Cytokine induction by LDL(−) in monocytes and lymphocytes occurs both at RNA and protein levels [15]. In a genomic study it was shown that LDL(−) modifies the transcription of other genes related to inflammation and atherosclerosis in mononuclear cells. Among these modifications, LDL(−) promotes Fas upregulation, colony stimulating factor 1 receptor (CSF1R), and CD36 downregulation [52]. Fas has been reported to be involved in apoptosis and in cytokine induction [53, 61]. Therefore, Fas induction could be related to these biological effects of LDL(−). 

#### 3.4.3. Increased Affinity to Proteoglycans

LDL(−) presents higher affinity to PG than LDL(+). Aggregation of LDL(−), mediated by its PLC-like activity, seems to be important in its binding to PG since agLDL(−) is the LDL(−) subfraction that has the highest affinity to PG [12]. It has been hypothesized that alterations in the N-terminal extreme of apoB could be responsible for this increased binding [12]. LDL(−) could also act as a seeding factor since its aggregation stimulates aggregation of native lipoproteins. This process could promote the subendothelial retention of lipoproteins in vivo. The higher LDL(−) binding to PG and subendothelial retention could favor LDL(−) exerting its inflammatory action locally in the microenvironment of the arterial wall, besides acting on circulatory cells. Moreover, LDL(−) retention in the arterial intima would allow induction of cytokine release for a longer period of time.

#### 3.4.4. Global Inflammatory Effect of LDL(−)

It is noteworthy that LDL(−) promotes an inflammatory action on several cell types that participate in the atherosclerotic process. The biological effects found in “in vitro” experiments with a cell type cannot be considered individually because in a physiological context all the cell types interact. These interactions enhance the effect promoted by LDL(−) since some cytokines can induce the release of other cytokines, and, moreover, cytokines induced in a cell type can act on other cell types, as shown in Figure 2. 

LDL(−) in the circulation induces cytokine release in monocytes and lymphocytes. LDL(−) also promotes chemokine and adhesion molecule expression in endothelial cells, and these molecules promote the recruitment of circulatory leukocytes to endothelium. In addition, cytokine released by endothelial cells can act on cells that are already in the subendothelial space, such as recruited monocytes, macrophages, and smooth muscle cells. These cell types are also exposed to LDL(−) retained in the subendothelial space by PG. In addition, LDL(−) retained in the arterial wall could be further modified by oxidation since it is not protected by the plasma antioxidants and by enzymatic hydrolysis. These modifications of LDL(−) could lead to additional inflammatory actions on cells or to further aggregation of LDL(−). This latter effect could favor LDL(−) recognition by SR, promoting the formation of foam cells.

The biological effects described for LDL(−) are, in part, similar to that for mmLDL/oxLDL, whose involvement in the atherosclerotic process has been extensively reported. Nevertheless, there are several differences between the biological properties of these modified LDLs, shown in Table 1.

## 4. An Antiatherogenic LDL? 

Early observations regarding the cytotoxic effect of LDL(−) on endothelial cells typecasted this modified LDL as a “bad guy” in the atherosclerotic process. Further findings describing an apoptotic and inflammatory effect for LDL(−) also supported this idea. However, in recent years, other studies ascribed some putative anti-inflammatory and regulatory properties to LDL(−), questioning whether LDL(−) is really so “bad”. 

The main modulatory property promoted by LDL(−) is the induction of the anti-inflammatory cytokine IL10 in monocytes and lymphocytes. The relationship between IL10 and protection against atherosclerosis has been widely established in human clinical studies and in mice [62, 63]. The protective role of IL10 has also been demonstrated in studies with cultured cells, in which IL10 regulates the production of proinflammatory cytokines [64]. All data support a physiological function of IL10 as a controller of inflammatory response, as it seems to be the role of IL10 induced by LDL(−). IL10 diminishes the release of the inflammatory cytokines promoted by LDL(−) in monocytes and lymphocytes [15]. The addition of exogenous IL10 and blocking of IL10 action with antibodies, respectively, inhibit and increase the cytokine release promoted by LDL(−). Therefore, if LDL(−) does not induce IL10 in mononuclear cells, its inflammatory response will be higher. IL10 also promotes its inhibition by negative feedback to avoid the absence of an inflammatory response [15]. Taken together, these data show that LDL(−) counteracts its inflammatory cytokine induction in leukocytes through IL10 to avoid an excessive inflammatory response. Otherwise, this counteracting mechanism does not occur in endothelial cells because they do not produce IL10 in response to LDL(−) [59]. 

Another modulatory action promoted by LDL(−) is the induction of nuclear translocation of the transcription factor Nrf2 in macrophages [50]. Nrf2 decreases apoptotic activity and modulates the metabolic response to oxidative stress. Accordingly, LDL(−) promotes cell survival and adaptation to oxidative stress in macrophages and endothelial cells [65]. Nrf2 production by LDL(−) in macrophages attenuates their LDL(−)-induced apoptosis [50]. IL10 production by LDL(−) could also be involved in the regulation of apoptosis since IL10 promotes antiapoptotic effects in macrophages [66]. However, Nrf2 activation does not overcome the proapoptotic effect of LDL(−), and IL10 induction does not avoid inflammatory cytokine release either. These compensatory mechanisms could limit the atherogenic effects of LDL(−) but could not inhibit them altogether. 

A study by Bancells et al. showed that LDL(−) could avoid monocyte differentiation to macrophages [52], in contrast to oxLDL [67, 68]. LDL(−) downregulates the expression of molecules involved in monocyte differentiation: CSF1R, CD36, and peroxisome proliferator-activated receptor *γ* (PPAR*γ*) [52]. The inhibition of PPAR*γ* by LDL(−) could promote the CD36 downregulation since PPAR*γ* is a transcription factor that induces CD36 expression [69]. In contrast to these results, Pedrosa et al. observed that LDL(−) induces CD36 in macrophages [50]. On the other hand, it has been described that LPS downregulates the expression of CD36 and CSF1R in inflammatory situations, hindering excessive cell activation [70]. 

It has been proposed that the combination of PAF-AH and phospholipase C-like enzymatic activities associated with LDL(−) could play a role in the inactivation of oxidized phospholipids (oxPL), inflammatory components of oxLDL, and mmLDL [6]. PAF-AH activity hydrolyzes PAF-like phospholipids, which could prevent LDL oxidation, but it yields LPC that is an inflammatory molecule. Therefore, LPC could be hydrolyzed by the PLC-like activity of LDL(−) since it is the main substrate. According to this theory, LDL(−) develops a protective function since it avoids the presence of oxLDL or mmLDL, which have greater atherogenic effects than those of LDL(−) [6]. 

Finally, the most recent observation showing an anti-inflammatory action for LDL(−) is the counteraction of LPS-induced inflammation in monocytes [16]. This counteracting action of LDL(−) seems to be a consequence of the competition between LPS and LDL(−) for the same pathway in monocytes. Both LPS and LDL(−) promote cytokine release in monocytes through the activation of two receptors, CD14 and toll-like receptor 4 (TLR4) [16]. This observation suggests a putative protective action of LDL(−) by decreasing systemic LPS toxicity in cases of overwhelming inflammation, such as a sepsis syndrome arising from bacterial infection.

There is controversy regarding a putative competition between modified LDLs and LPS. Some authors describe an inhibitory action of oxLDL on the LPS effect in monocytes [71, 72]. In contrast, others have reported that native LDL [73] and oxLDL [74] present a synergic proinflammatory effect on monocytes when incubated with LPS. These discrepancies are probably related to the concentrations of LPS and LDL and to the type and degree of LDL modification. OxPL have been described to compete with LPS in the inflammatory effect [75]. In spite of TLR4 binding to small amounts of oxPL [76], oxPL are considered weak agonists for TLR4. The most accepted idea is that oxPL could inhibit TLR signaling by preventing LPS interaction with accessory proteins involved in TLR4 binding [75, 77, 78]. In the atherosclerotic lesion there could be oxPL and mmLDL. However, their presence in plasma is not so feasible, whereas circulating LDL(−) is a likely physiological TLR-ligand. 

## 5. Molecular Mechanisms Involved in LDL(−) Effect on Cells

As reviewed above, several LDL(−) actions on cells have been described. Nevertheless, the components or the physico-chemical characteristics of LDL(−) responsible for its effect on cells are not totally understood. The receptors that bind and mediate the biological effects of LDL(−) are reasonably well established, but the intracellular pathways activated by LDL(−), which would lead to its inflammatory and anti-inflammatory effects on cells, are not well known. 

### 5.1. Inflammatory Components of LDL(−)

Some authors suggest that oxidation is the mechanism responsible for the inflammatory and cytotoxic effects of LDL(−) [13, 14]. Other authors do not attribute an oxidative origin to LDL(−) [20] and do not find a cytotoxic effect either [7, 15]. They suggest other explanations for the atherogenic properties of LDL(−), such as the increased content in LPC, NEFA, and CER.

The increased PAF-AH activity associated with LDL(−) [10] might be the origin of the increased amount of LPC and NEFA in LDL(−). Both components are involved in the cytokine release promoted by LDL(−) in endothelial cells [8]. The increased NEFA content of LDL(−) is also involved in the induction of cytokine release promoted by LDL(−) in monocytes [26]. In these cells, the presence of HDL caused a diminution in both the NEFA content in LDL(−) and the cytokine release induced by LDL(−) [26], thereby supporting a relationship between NEFA and inflammation promoted by LDL(−).

PLC-like activity of LDL(−) seems to be involved in the cytokine release promoted in monocytes through the generation of CER. PLC-like activity, CER content, and cytokine release are reduced by preincubation of LDL(−) with HDL, suggesting a relationship between these LDL(−) properties [26]. PLC-like activity hydrolyzes the polar head of choline-containing phospholipids and preferentially degrades LPC, with intermediate medium efficiency for sphingomyelin (SM) and with lower efficiency for phosphatidylcholine (PC). The products of this hydrolysis are CER, monoacylglycerol (MAG), diacylglycerol (DAG), and phosphorylcholine (Pchol). Pchol is water soluble and presumably leaves the LDL particle, but the other products are hydrophobic and remain retained in the LDL particle. Even though LPC is rapidly degraded by the PLC-like activity, MAG would be scarce in LDL since the amount of LPC is much lower (2-3% of total phospholipids in LDL) than PC (70%) and SM (20%). For this reason, CER and DAG are more abundant products of PLC-like activity than MAG in LDL(−). CER and DAG are considered as bioactive and inflammatory molecules that promote cell signal transduction. A relationship between PLC-like activity and increased CER and DAG content in LDL(−) has been shown. The involvement of CER content in LDL, but not of DAG, in cytokine release in monocytes has been demonstrated [9]. 

The role of CER and NEFA in the cytokine release promoted by LDL(−) in monocytes could be explained by the fact that both compounds can bind to CD14 [79]. It is well known that CD14 binds to inflammatory ligands and afterwards interacts with TLR4 to mediate cytokine release. However, apart from CER and NEFA, other factors seem to contribute to the inflammatory effects of LDL(−). LDL modified “in vitro” to increase its content of CER or NEFA to a similar or higher degree than LDL(−) promotes a lower inflammatory action than LDL(−). This suggests that a combination of several LDL(−) properties contributes to its inflammatory effect.

LDL(−) presents a higher aggregation level than LDL(+), probably as a consequence of its increased CER and NEFA content. However, the high aggregation of LDL(−) as a cause of its inflammatory properties has been ruled out. In vitro aggregation of LDL does not promote cytokine release in monocytes compared to native LDL [9]. But as discussed previously, aggregation is responsible for the increased binding to PG of LDL(−), where it would remain retained favoring its inflammatory action.

### 5.2. LDL(−) Cell Receptors

The first step in the knowledge of the mechanisms involved in the biological effects for LDL(−) is to determine the receptor or receptors that recognize LDL(−) and mediate the starting signals in the activation of intracellular pathways. Several physicochemical properties ascribed to LDL(−), such as electronegative charge, higher aggregation level, conformational changes in apoB, and increased content in inflammatory lipids, suggest that LDL(−) interacts with different cell receptors than LDL(+). This would influence the clearance of LDL(−) from the circulation and the activation of certain intracellular pathways involved in the induction of cytokine release promoted by LDL(−). 

Early studies regarding cell binding focused on LDL receptor (LDLr). LDL binds to LDLr through its apoB lysine residues. As LDL(−) has a higher negative charge than LDL(+), it was expected that LDL(−) would bind to LDLr with lesser affinity. The first study performed in this regard observed that LDL(−) presented loss of affinity for LDLr [4]. These results concur with those of Benitez et al. who found that LDLr affinity was 3-fold lower for LDL(−) than for LDL(+) [80]. The lower affinity for LDLr could be partly explained by the higher NEFA content in LDL(−) [80], its increased degree of aggregation [27], and the abnormal conformation of its apoB [12]. The global consequence of the loss of affinity would be a diminished clearance of LDL(−) from plasma circulation, making this particle susceptible to further modifications. In contrast, other studies reported that LDL(−) binds to LDLr with a similar or increased affinity compared to LDL(+) [13, 19, 81]. The increased binding was attributed to the increased content in apoE of LDL(−). 

As LDL(−) possesses an electronegative charge, some SR could uptake this subfraction, as occurs in the case of other modified LDL, such as oxLDL or acetylated LDL [82]. Once again, there is no concensus on this point as some authors describe no differences in the uptake through type A SR [4, 80, 83] while others suggest that LDL(−) could be recognized by SRs [84, 85]. In any case, LDLr and SR should not be related to cytokine release but to plasma cholesterol uptake and accumulation of intracellular cholesterol, respectively. So which cell receptor or receptors are involved in the inflammatory effects of LDL(−)?

Chen et al. suggested that the PAF receptor plays a role in mediating apoptotic effects of L5 in endothelial cells [44]. However, as LDL(−) presents high PAF-AH activity [10], its PAF content can be expected to be low. More recently, Chen and coworkers also reported that lectin-like oxidized LDL receptor (LOX-1) plays a role in L5 recognition. As a consequence of binding to LOX-1, L5 induces several biological effects in endothelial cells, including apoptosis and LOX-1 upregulation [46, 48, 54]. LOX-1 is the main SR in endothelial cells, whereas low LOX-1 expression can be found in monocytes [86]. Moreover, oxLDL, the typical ligand for LOX-1, does not compete with LDL(−) for its binding to monocytes [16]. For these reasons, it is unlikely that LOX-1 is the mediator of the cytokine release promoted by LDL(−) in monocytes. Other SRs, such as SRA, are expressed in low amounts in monocytes, increasing its expression during the differentiation of this cell type to macrophages.

The involvement of TLRs in the biological effects of LDL(−) had been suggested [87] and recently demonstrated [16]. TLRs are immune response receptors against pathogens, which are related to atherosclerosis [88]. TLR ligands, such as LPS, bind to CD14, a differential marker of monocytes, which associates with TLR2 or TLR4 to induce intracellular signal transduction [89]. TLR2 and TLR4 can bind directly to LPS and also modified lipoproteins. The activation of the system CD14-TLR4 by mmLDL has been studied in depth by Miller and coworkers, particularly in macrophages. They found that CD14 binds to mmLDL, the binding site being different from that for LPS [90]. This binding promotes CD14 and TLR4 association and leads to stimulation of phagocytosis [90], macropinocytosis, and cholesterol accumulation [91]. mmLDL also induces inflammatory cytokines in macrophages, such as MCP1, IL6, and tumor necrosis factor *α* (TNF*α*), in a TLR4-dependent or -independent manner [92]. Studies by Chávez-Sánchez et al. show that, in monocytes and macrophages, mmLDL induces IL1, IL6, IL10, and TNF*α* secretion through CD14, TLR4, and TLR2 [93, 94]. Other authors have reported that oxLDL promotes MCP1 and IL8 release and upregulates TLR4 in monocytes [95], and mmLDL also induces TLR4 in macrophages [96]. Because of the role of CD14-TLR4 in the inflammatory action of mmLDL, the involvement of TLRs in the LDL(−) effects on cells seems to be feasible. According to this, recent findings from our group have demonstrated that CD14 is the main receptor of LDL(−) in monocytes. CD14 association with TLR4 triggers the subsequent intracellular machinery leading to cytokine release [16]. The fact that LDL(−) shares the CD14-TLR4 pathway with LPS explains the previously mentioned cross-competition between LDL(−) and LPS in binding to monocytes and in cytokine release. 

### 5.3. Intracellular Mechanisms Activated by LDL(−)

Knowledge about intracellular signaling pathways activated by LDL(−) that lead to cell response is scarce. In contrast, the activation of several signaling pathways by mmLDL is better known, particularly in macrophages. Some of these pathways could also be activated by LDL(−).

In macrophages, mmLDL activates phosphoinositide-3-kinase (PI3k) by TLR4-dependent or -independent pathways, [90, 92] initiating Akt signaling [92]. It has also been suggested that LDL(−) activates PI3k and nuclear factor *κ*B (NF*κ*B) in cardiomyocytes leading to induction of apoptosis [51]. However, these findings contrast with those reported for the electronegative L5 subfraction in endothelial cells and endothelial progenitor cells, where the PI3k-Akt pathway is inhibited via LOX-1 [46, 48, 54]. As endothelial progenitor cells derive from circulating monocytes, LDL(−) could also have an inhibitory effect on the PI3k-Akt pathway in monocytes. 

It has been described that mmLDL induces the recruitment of spleen tyrosine kinase to TLR4 in macrophages [91, 97, 98]. This leads to phosphorylation of endothelial cell signal-regulated kinase (ERK1/2) and of c-Jun N-terminal kinase, which finally induces activating-protein 1 (AP1) [98]. In endothelial cells, the stimulation of TLR4 by oxLDL is described to induce the activation of ERK and p38 mitogen-activated protein kinase [99]. The involvement of these kinases on the biological effects of LDL(−) has not yet been studied.

Several observations show that AP1 and NF*κ*B seem to be involved in the inflammatory effects of LDL(−). In HUVEC, an increased nuclear translocation of some components of these transcription factors was observed (p65 and p50 for NF*κ*B and c-jun, cfos, and ATF2 for AP1) [100]. AP1 and NF*κ*B have also been reported to be involved in VCAM induction by LDL(−) [57]. A gene expression study in leukocytes suggests the activation of NF*κ*B and downregulation of PPAR*γ* [52]. The involvement of NF*κ*B and AP1 activation in the inflammatory effect of LDL(−) in monocytes has also been recently reported [16]. 

## 6. Physiological Effects of LDL(−)

It is difficult to ascertain the physiological effects that LDL(−) could exert in vivo, where other factors can contribute to modify its action on cells. The role displayed by LDL(−) will probably depend on the cell environment in each particular situation. The presence of other lipoproteins or cell activators, such as HDL and LPS, could modulate the biological action of LDL(−). Moreover, LDL(−) can promote different biological effects depending on the cell type. For example, LDL(−) downregulates CD36 expression in monocytes, probably to inhibit activation of these cells and differentiation to macrophages [52]. In contrast, LDL(−) upregulates CD36 in macrophages [50] to eliminate toxic compounds, including oxidized lipids, leading to foam cell formation. 

**Figure 3:**
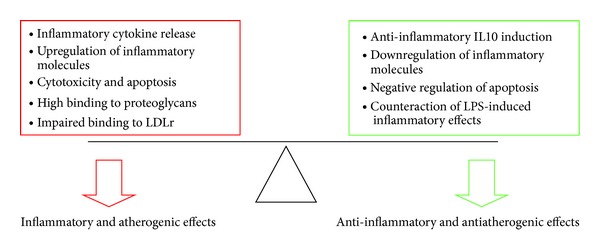
Balance of inflammatory and anti-inflammatory effects of LDL(−) on cells. LDLr: LDL receptor, IL10: interleukin 10, LPS: lipopolysaccharide.

The fact that LDL(−) is recognized by innate immune receptors on monocytes suggests, a priori, that it could be a “self-pathogen” particle that the immune system has to eliminate. This is supported by the detection of antiLDL(−)-autoantibodies and immunocomplexes [39]. Although some anti-inflammatory actions on cells have been ascribed to LDL(−), the abundant atherogenic properties would lead to a global inflammatory effect rather than to an atheroprotective effect, as shown in Figure 3. Probably, it would be more appropriate to consider the anti-inflammatory actions described for LDL(−) as regulatory/modulatory mechanisms to minimize the inflammatory effect of this modified LDL. 

**Figure 4:**
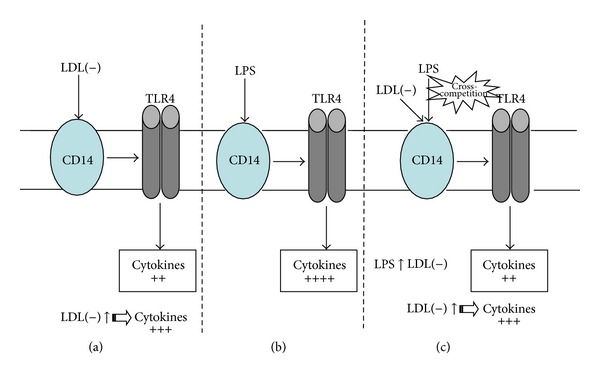
Cytokine induction through CD14-TLR4 by LPS and LDL(−). In the absence of bacterial infection, CD14-TLR4 activation mediated through LDL(−) triggers an inflammatory response that would be deleterious in case of high LDL(−) concentration (a). This effect would be lower than that induced by LPS at high concentrations (infection) in the absence of LDL(−) (b). When LPS and LDL(−) coexist, there is a competition between the two stimuli. The global effect will depend on the relative concentration of both molecules (c). TLR4: toll-like receptor 4, LPS: lipopolysaccharide, LDL(−): electronegative LDL.

Thus, the classification of the biological effect of LDL(−) as positive or negative is not so categorical since it would depend on the situation. Cytokine release promoted by LDL(−) could be considered as an atherogenic action, but, in turn, this inflammatory response would be beneficial in counteracting an external aggression. Regarding the physiological role of LDL(−)-induced apoptosis, it is not so clear whether this is an atherogenic effect. Apoptosis could be considered detrimental in late atherosclerotic lesions, but, in early atherosclerotic lesions, the clearance of apoptotic cells is associated with decreased lesion progression [101]. Therefore, these two “atherogenic” properties may not be so bad, and, only when these processes are uncontrolled or excessive, they became detrimental. On the other hand, a putative protective action may not be so good. The counteraction by LDL(−) of the LPS-induced inflammatory effect could be protective. Nevertheless, LDL(−) exerts an inflammatory action that could also be harmful when LDL(−) concentrations increase, even though it is less deleterious than LPS, as shown in Figure 4. 

LDL(−) could play a role as a modulator of the inflammatory response to avoid detrimental and inappropriate immune responses. The proportion of LDL(−) is increased in inflammatory situations, such as rheumatoid arthritis or DM. In such events, it could modulate the immune response to some degree. It can be hypothesized that LDL(−) would emerge as a negative feedback to counteract an excessive/overwhelming inflammatory response and play a protective role. It thus seems likely that LDL(−) is more of a consequence of inflammatory situations than a cause. 

## 7. Conclusions

In summary, LDL(−) is a heterogeneous modified LDL which promotes several inflammatory actions on cells. LDL(−) also promotes some anti-inflammatory actions to control an excessive inflammatory response. The global effect of LDL(−) will be the result of the combination of its inflammatory/anti-inflammatory properties. The importance of each individual property in the global action of LDL(−) depends on the physicochemical characteristics of LDL(−) and the cell milieu. Taken together, all data concur that, depending on the context, LDL(−) promotes or inhibits inflammation, playing a dual role in atherogenesis. 

## Figures and Tables

**Table 1:** Differences in the properties of oxLDL/mmLDL and LDL(−).

oxLDL/mmLDL	LDL(−)
(i) Oxidized particle	(i) Resistance to oxidation. Oxidized LDL?
(ii) 0.1–0.5% of total plasma LDL	(ii) 3–5% of total plasma LDL (increased in some pathologies)
(iii) No increased PG affinity	(iii) Increased PG affinity
(iv) No phospholipolytic activity	(iv) Associated phospholipolytic activities
(v) Recognition by SRA, EC accumulation	(v) No recognition by SRA, no EC accumulation
(vi) TNF induction, no IL10 induction	(vi) No TNF induction, IL10 induction.
(vii) CD36 upregulation and PPAR*γ* upregulation	(vii) CD36 downregulation (and PPAR*γ*) in monocytes, CD36 upregulation in macrophages
(viii) Cytotoxicity	(viii) Discrepances in cytotoxic effect
(ix) No induction of LDL fusion	(ix) Induction of LDL fusion
(x) Altered immunoreactivity to antibodies anti-apoB	(x) Altered immunoreactivity to antibodies anti-apoB, but different than oxLDL
(xi) No competition with LDL(−) for binding to monocytes	(xi) No competition with oxLDL for binding to monocytes, competition with LPS

(oxLDL/mmLDL) and LDL(−). oxLDL: oxidized LDL, mmLDL: minimally modified LDL, PG: proteoglycans, SRA: type A scavenger receptor, TNF*α*: tumor necrosis factor *α*, IL10: interleukin 10, EC: esterified cholesterol, PPAR*γ*: peroxisome proliferator-activated receptors, LPS: lipopolysaccharide.
